# Melatonin as an anti-inflammatory hormone bridging migraine relief and cancer immunity enhancement: a literature review

**DOI:** 10.3389/fimmu.2025.1644066

**Published:** 2025-07-28

**Authors:** Qian Zhu, Jin Yang, Jieying Zhang, Qian Song, XinXin Zhang, Menghan Li, Menglong Zhang, Lei Shi, Xiaoli Song

**Affiliations:** ^1^ National Clinical Research Center for Chinese Medicine Acupuncture and Moxibustion, Tianjin, China; ^2^ First Teaching Hospital of Tianjin University of Traditional Chinese Medicine, Tianjin, China; ^3^ Center for Acupuncture in Brain Disease Treatment, Jinjiang Hospital of Traditional Chinese Medicine, Quanzhou, China

**Keywords:** melatonin, anti-inflammatory, migraine, cancer immunity, tumour micro-environment, neurogenic inflammation

## Abstract

Melatonin, once relegated to the circadian periphery, has resurfaced as a pleiotropic immunomodulator capable of quelling neurogenic inflammation while invigorating antitumour defence. Migraine—and its disabling, episodic neurovascular pain—shares an “inflammatory genome”, defined here as a conserved danger-response gene set (NF-κB, NLRP3, IL1B, NOS2), with the metabolic chaos that subdues cytotoxic immunity in solid cancers; both ignite NF-κB, NLRP3 and reactive-oxygen cascades that erode tissue homeostasis. Emerging evidence shows that endogenous melatonin declines precede migraine attacks, and nightly supplementation rivals first-line preventives in shrinking monthly headache burden while restoring sleep architecture. In parallel, supraphysiological pulses re-programme tumour-associated macrophages toward an iNOS-rich M1 phenotype, amplify granzyme-B output from CD8^+^ T cells and down-tune PD-L1 expression on malignant and myeloid cells, thereby widening the therapeutic window of immune-checkpoint blockade. The same cytokines suffused during a migraine flare—IL-6, TNF-α, ROS—subvert antitumour surveillance; melatonin extinguishes these mediators, synchronises clock-gene–driven metabolism and stitches a biochemical thread between headache relief and cancer immunity. Nanocarrier formulations, chronobiology-guided dosing and rational combinations with CGRP inhibitors or PD-(L)1 antibodies are already advancing through translational pipelines. This review distils molecular pharmacology, pre-clinical models and early-phase trials to portray melatonin as a single, evolutionarily conserved molecule that orchestrates bilateral protection across nervous and oncologic frontiers. By integrating chronotherapy, immunology and neurovascular biology, we aim to identify diagnostic blind spots, repurpose therapeutics and chart a roadmap toward precision strategies that simultaneously alleviate migraine disability and fortify antitumour immunity.

## 1 Introduction

Migraine and cancer appear to occupy disparate clinical arenas—one an episodic neurovascular pain syndrome, the other a relentless proliferative disorder—yet both are deeply rooted in dysregulated inflammatory signalling. Melatonin, an indoleamine chiefly synthesized in the pineal gland but also produced locally in immune and vascular tissues, has emerged as a potent endogenous brake on such inflammation. Beyond synchronizing circadian rhythms, melatonin dampens nuclear-factor-κB and NLRP3-inflammasome activation, scavenges reactive oxygen species, and tunes cytokine profiles toward an anti-inflammatory, interferon-γ–rich milieu (1–3). These broad immunomodulatory actions nominate the hormone as a molecular hinge linking migraine relief with the enhancement of anticancer immunity.

Compelling evidence implicates melatonin deficiency in migraine pathophysiology. Cross-sectional analyses reveal lower nocturnal and salivary melatonin levels in episodic and chronic migraineurs compared with matched controls, correlating inversely with attack frequency (4). Small-to-moderate randomized trials have shown that nightly melatonin (2–5 mg) rivals amitriptyline or topiramate in reducing monthly headache days, while conferring superior tolerability and sleep restoration (5, 6). Mechanistic work in trigeminovascular models demonstrates that melatonin, via MT2 receptors expressed on dural vessels and trigeminal ganglion neurons, suppresses calcitonin gene-related peptide (CGRP) release, stabilizes mast-cell degranulation, and curtails neurogenic plasma extravasation—hallmark events in migraine initiation (7, 8). Collectively, these findings position melatonin not as a mere chronobiotic but as an active anti-inflammatory neuromodulator within the migraine circuit.

An equally expansive literature now documents melatonin’s capacity to re-shape the tumour micro-environment. In pre-clinical glioma, melanoma, and colorectal cancer models, exogenous melatonin re-polarizes tumour-associated macrophages toward an M1 phenotype, boosts cytotoxic granzyme-B production by CD8^+^ T cells, and down-regulates programmed-death ligand-1 (PD-L1) on malignant and myeloid cells, thereby amplifying responses to anti-PD-1 therapy (9–11). Parallel studies in human breast and hepatocellular carcinoma show that melatonin inhibits hypoxia-inducible factor-1α, vascular endothelial growth factor, and matrix metalloproteinase-9, signalling axes that also participate in trigeminovascular sensitization (12, 13). These convergent targets suggest that the hormone’s anti-inflammatory portfolio extends seamlessly from neuronal to oncologic contexts.

Notably, the same cytokines and danger signals elevated during a migraine attack—interleukin-6, tumour-necrosis factor-α, and ROS—are drivers of tumour immune evasion and angiogenesis (14, 15). Melatonin’s ability to quench these mediators while restoring circadian regulation of immune checkpoints offers a unifying framework: by damping neurogenic inflammation it can alleviate migraine pain, and by recalibrating innate and adaptive immunity it can fortify antitumor defences.

This review synthesizes contemporary evidence—from molecular pharmacology to clinical trials—on melatonin as an anti-inflammatory hormone bridging migraine relief and cancer immunity enhancement. We first outline canonical melatonin signalling and its anti-inflammatory mechanisms, then interrogate its roles in migraine modulation and oncologic immunity, before considering translational opportunities and research gaps. In doing so, we aim to illuminate how a single, evolutionarily conserved molecule can orchestrate multidimensional protection across seemingly disparate disease states.

## 2 Melatonin signalling and anti-inflammatory mechanisms

**Figure 1:**
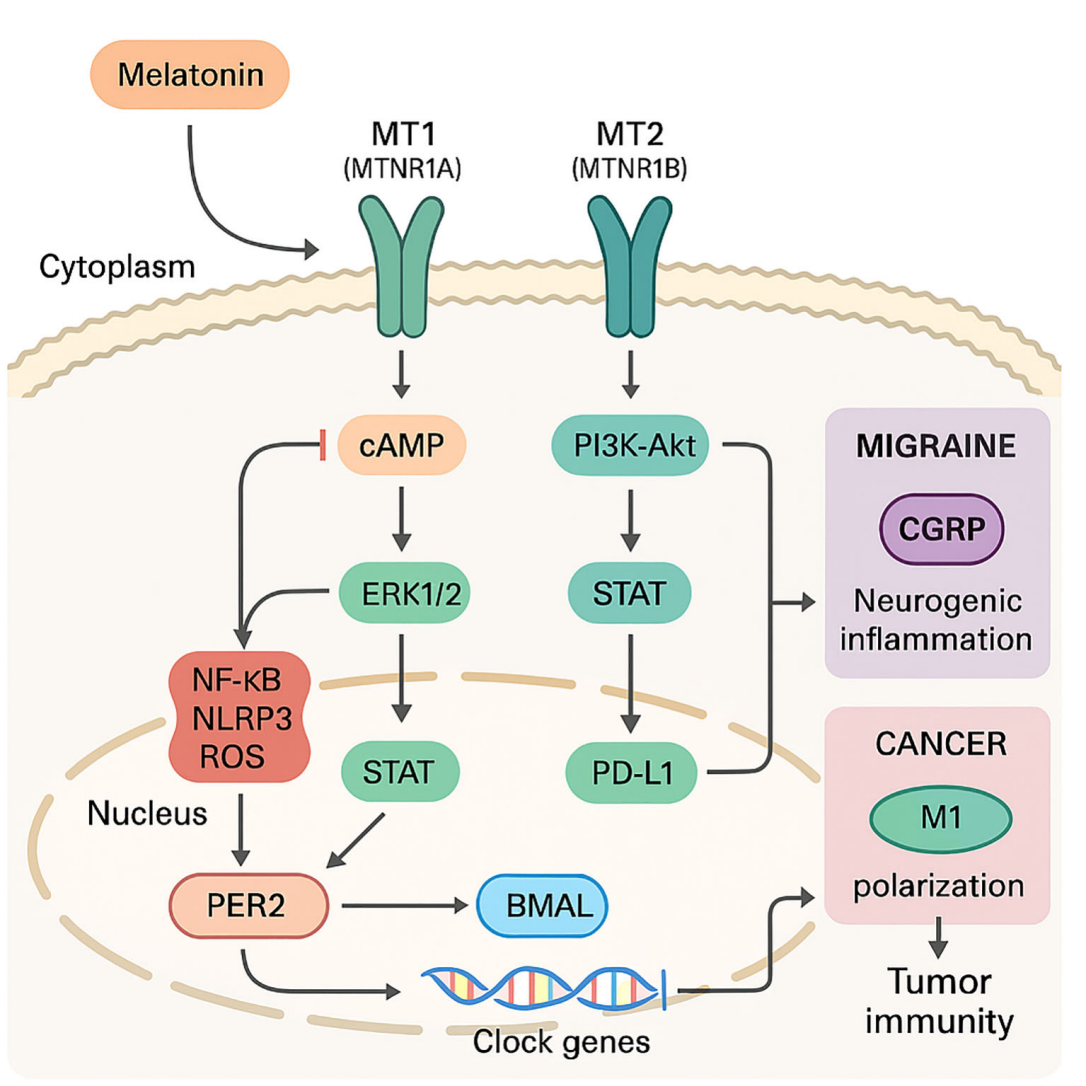
Melatonin receptor signalling cascade linking migraine suppression and cancer immunity.

As shown in **Figure 1**, melatonin engages two high-affinity G-protein–coupled receptors, MT1 (encoded by MTNR1A) and MT2 (MTNR1B), that are widely expressed on neuronal, vascular, and innate-immune cells. MT1 predominantly signals through Gi-cAMP suppression and ERK1/2, favouring neurovascular vasoconstriction and CGRP restraint, whereas MT2 more strongly couples to PI3K-Akt-Nrf2 and STAT pathways that reprogramme macrophages and down-regulate PD-L1 in tumours. Ligand binding preferentially recruits Gi/Go proteins, lowering cAMP, while biasing downstream effectors such as ERK1/2, PI3K–Akt, and PKC toward cytoprotective outputs (16, 17). Within seconds, these cascades converge on clock genes (PER2, BMAL1) and retinoid-related orphan receptors (RORα/γ), weaving circadian timing into inflammatory tone (18, 19). Melatonin lengthens PER2 nuclear dwell time while enhancing BMAL1 acetylation, sharpening the amplitude of immune-gene oscillations that rejuvenate exhausted T and NK cells. Lipophilicity allows melatonin to accumulate in mitochondrial membranes, where by binding cardiolipin it stabilises complex I–IV supercomplexes, thereby reducing electron leak and directly scavenging peroxynitrite and hydroxyl radicals—actions that blunt danger-signal generation at its source.

Receptor activation rapidly tilts canonical inflammatory hubs toward resolution. In dextran-sodium-sulfate colitis, an archetype of mucosal inflammation, MT2 ligation triggers a PI3K–Akt–Nrf2–SIRT1 relay that simultaneously lifts antioxidant gene transcription and blocks IκBα degradation, preventing nuclear translocation of NF-κB p65 and the transcription of Il1b, Tnf, and Nos2 (20, 21). The same circuit represses RORα-dependent inflammasome priming, highlighting how circadian and immune signals intersect under melatonergic control.

A complementary brake operates through the deacetylase SIRT1. By deacetylating both p65 and the FOXO family, SIRT1 lowers NF-κB DNA binding and enhances oxidative-stress defences (22, 23). In diabetic-retinopathy models, pharmacological blockade of SIRT1 erases the antioxidant and anti-cytokine benefits of melatonin, underscoring a direct hormonal dependency on this epigenetic switch. Key SIRT1 targets include p65, p53 and FOXO3a in migraine-linked neurons, and HIF-1α and c-Myc in tumour cells, situating the enzyme at the nexus of energy sensing and inflammatory restraint.

At the level of danger-signal sensing, melatonin disarms the thioredoxin-interacting-protein (TXNIP) gate that licenses NLRP3 inflammasome assembly (24, 25). In endotoxin-challenged tissues the hormone suppresses ER-stress–driven TXNIP accumulation, thereby curtailing caspase-1 activation and the downstream release of IL-1β and IL-18—cytokines that fuel both neurovascular pain and tumour immune escape (26).

These molecular programmes scale upward to reshape innate-immune phenotypes. In the central nervous system melatonin, via MT1-linked JAK2–STAT3 signalling, converts microglia from a pro-inflammatory M1 state to an M2 reparative profile, reducing nitrosative stress and fostering axonal myelination (27, 28). Peripherally, the hormone reverses the arginase-1–dominant metabolism of tumour-associated macrophages, reinstating iNOS activity and nitric-oxide production that favour cytotoxic immunity over wound-healing fibrosis (8, 29, 30).

These receptor-dependent and receptor-independent mechanisms position melatonin as a master rheostat that synchronises redox balance, inflammasome restraint, and innate-cell programming. Such broad anti-inflammatory leverage not only dampens the sterile neurogenic flare that precipitates migraine but also restores immunological vigour within the tumour micro-environment.

## 3 Melatonin in migraine relief: neurovascular and immune modulation

Melatonin receptors are strategically positioned along the trigeminovascular axis that drives migraine pain. Immunohistochemical mapping reveals MT2‐rich varicosities on dural arteries, meningeal fibroblasts, and >60% of small-diameter trigeminal-ganglion neurons, providing direct molecular “sockets” through which the hormone can influence both vascular tone and nociceptive firing (31, 32). At the neurovascular interface, nanomolar melatonin abrogates calcitonin-gene-related peptide (CGRP)–evoked dilation of middle cerebral and meningeal arteries and curtails the concomitant rise in cyclic-AMP, an effect lost after MT2 blockade (33, 34). This vaso-normalising action limits the shear-stress signals that normally amplify CGRP release, thereby short-circuiting the feed-forward loop that initiates migraine aura and throbbing pain.

Parallel control is exerted over meningeal immunity. In ex vivo dura mater, melatonin suppresses CGRP-triggered mast-cell degranulation by blunting PLC-β activation and subsequent Ca²^+^ influx, thereby curtailing the efflux of histamine and tryptase—mediators that otherwise prime nociceptors and open the blood–meningeal barrier. These effects depend on MT2 coupling to the PI3K–Akt–Nrf2 axis, linking antioxidant defence to neurogenic-inflammation restraint (20, 35). Within the trigeminal nucleus caudalis, the hormone polarises resident microglia away from an inducible-nitric-oxide-synthase–high M1 phenotype toward an arginase-1–expressing M2 state, diminishing IL-1β and TNF-α release and dampening central sensitisation. This transition is reinforced by melatonin-induced IL-10 and TGF-β secretion, which up-regulate Arg-1 and suppress iNOS to consolidate the M2 programme. Experimental MT2 knock-down abolishes this switch, underscoring a receptor-locked mechanism.

Systemically, melatonin lowers iNOS activity, nitrite/nitrate accumulation, and IL-1β secretion in peripheral blood mononuclear cells obtained from migraineurs, suggesting that nightly supplementation can re-balance pro- and anti-inflammatory cytokine tone even outside the central nervous system (36, 37). These mechanistic insights align with clinical observations. Evening and nocturnal serum-melatonin concentrations inversely correlate with Migraine Disability Assessment scores, and patients in the lowest tertile exhibit a two-fold higher monthly attack rate. Randomised trials summarised in a recent systematic review show that 3 mg immediate-release melatonin reduces monthly headache days by ~2.7 compared with placebo and matches amitriptyline 25 mg for efficacy while causing fewer adverse events and modest weight loss (38–40).

Melatonin orchestrates a multi-layered defence, normalising meningeal haemodynamics, stabilising mast cells, reprogramming microglia, and re-shaping systemic cytokine profiles that converges on the attenuation of neurogenic inflammation. These same signalling nodes reappear in the tumour micro-environment, foreshadowing the hormone’s capacity to recalibrate anticancer immunity.

## 4 Melatonin-driven enhancement of cancer immunity

Mounting evidence now positions melatonin as a multi-tiered immunorestorative that counteracts the metabolic and cytokine constraints imposed by solid tumours. In experimental models spanning gastric, breast and lung carcinomas, physiological-to-pharmacological concentrations (1 µM–1 mM) re-programme the tumour micro-environment (TME) by converging on three pressure points: repolarisation of innate immune sentinels, revitalisation of cytotoxic lymphoid effectors and de-repression of exhausted adaptive checkpoints (41, 42). Through these axes the indoleamine converts “cold”, macrophage-dominant niches into “hot”, T-cell–inflamed landscapes more amenable to immune-checkpoint blockade.

Within 24 h of systemic delivery, melatonin reduces STAT3 phosphorylation—thereby dismantling the immunosuppressive IL-10/VEGF loop that nurtures tumour immune evasion—and diminishes HIF-1α accumulation in tumour-associated macrophages (TAMs), tipping their transcriptional programme from an arginase-1/VEGF-rich M2 phenotype toward an iNOS/IL-12-high M1 state (43, 44). Gastric-cancer xenografts treated with a 20 mg kg^-1^ download exhibit a three-fold rise in intratumoural MHC-II^+^/CD86^+^ macrophages and a parallel drop in PD-L1 expression (45, 46), effects that are abolished by MT1/MT2 dual antagonism—underscoring a receptor-locked mechanism.

Beyond macrophages, melatonin fortifies natural-killer (NK) and neutrophil arms of innate immunity. In ageing murine hosts—the demographic most prone to immunosenescence—seven-day supplementation (10 mg kg^-1^, i.p.) doubles splenic NK-cell numbers, boosts CD107a degranulation and elevates IFN-γ release via a JAK3–STAT5–T-bet relay (47, 48). Parallel work in orthotopic pancreatic cancer reveals that pineal-derived melatonin increments the density of CXCR2^+^ “N1” neutrophils, augments neutrophil extracellular-trap formation and curbs metastatic seeding, highlighting a granulocytic dimension to its onco-immunology profile (49, 50). N1’ neutrophils are CD62L^low^ CXCR2^+^ TNF-α^high^ effectors that promote tumour lysis, whereas ‘N2’ counterparts are Arg-1^high^ VEGF-A^high^ cells that foster angiogenesis and immunosuppression. At the adaptive interface, melatonin attenuates PD-L1 up-regulation driven by either hypoxia or sub-ablative radio-frequency ablation. In murine hepatocellular and breast-cancer models, 50 µg ml^-1^ melatonin lowers tumoural PD-L1 by 40% and expands the CD8^+^/Treg ratio two-fold, thereby magnifying the tumour-regressing effect of anti-PD-L1 antibodies without amplifying immune-related toxicities (51, 52). Mechanistically, the hormone destabilises HIF-1α, curtails c-Myc occupancy at E-box motifs (CACGTG) within the CD274 promoter and hampers exosomal PD-L1 cargo loading—collectively lifting the synaptic brake on cytotoxic lymphocytes.

Melatonin’s canonical clock-gene targets (BMAL1, PER2) intersect with T-cell metabolic checkpoints, restoring rhythmic oxidative phosphorylation and IL-2 responsiveness in tumour-infiltrating CD8^+^ cells (53). Gastric-cancer spheroid assays demonstrate that nightly (but not daytime) melatonin pulses synchronise T-cell NAD^+^ oscillations, sharpen granzyme-B release and restrain tumour growth—an effect abrogated in BMAL1-knockout mice (54).

The immunogenic rewiring induced by melatonin extends to conventional cytotoxics and targeted inhibitors. In BRCA-mutant ovarian cancer, co-administration with olaparib accelerates DNA-damage accumulation, up-regulates CXCL10 and fosters dendritic-cell cross-priming, culminating in durable tumour control after drug withdrawal (55, 56). Likewise, in EGFR-mutant lung carcinoma, melatonin reverses tyrosine-kinase-inhibitor resistance by normalising myeloid-derived suppressor-cell (MDSC) metabolism and reinstating CD8^+^ infiltration (57).

Although large-scale trials remain pending, an umbrella meta-analysis of 21 randomised studies reports a pooled relative risk of 0.66 for one-year mortality when melatonin (10–40 mg nightly) is combined with radio-chemotherapy across multiple solid tumours (58–60). Notably, benefit correlates with on-treatment rises in serum IFN-γ and declines in IL-10, echoing the pre-clinical circuitry outlined above (61).

Melatonin exerts an immunological “gear-shift” across both innate and adaptive compartments—converting anergic TAMs into nitric-oxide traffickers, invigorating NK-cell cytotoxicity, recruiting N1-phenotype neutrophils, reinstating rhythmic CD8^+^ metabolism and disarming PD-L1-mediated checkpoints. These convergent actions dovetail with the anti-inflammatory pathways that mediate migraine relief, underscoring a unifying thesis: by restoring circadian immunocompetence, melatonin can simultaneously quell neurogenic pain and fortify anti-cancer defences.

## 5 Therapeutic perspectives and future directions

The therapeutic promise of melatonin now rests on three inter-locking pillars—chronobiology‐guided dosing, formulation engineering, and rational combination therapy—that together can translate its dual anti-inflammatory leverage into migraine control and cancer immunopotentiation.

Because pineal output peaks in the early hours of the night, exogenous melatonin given 30–60 min before habitual sleep onset produces supraphysiological yet circadian-concordant plasma levels that reinforce MT-receptor signalling while minimising next-day somnolence. Recent chronotherapy trials in oncology show that drug administration synchronised to endogenous clock phase enhances immunogenic cell-death signatures, augments interferon-γ–driven antigen presentation and attenuates myelosuppression, highlighting the clinical utility of timed delivery. Comparable logic applies to migraine: evening dosing has already proved superior to morning intake for reducing nocturnal cortical spreading depolarisation, and emerging actigraphy-guided algorithms can now personalise dose timing to the individual dim-light melatonin onset.

Conventional oral tablets yield highly variable bioavailability (15 – 33%), are subject to first-pass metabolism and achieve only low nanomolar cerebrospinal-fluid concentrations. Nanostructured lipid carriers, chitosan-coated liposomes and PLGA-based microspheres have each prolonged systemic half-life beyond 6 h and delivered micromolar drug levels to intracranial and tumoural compartments in rodent models without off-target toxicity. Such vehicles not only widen the therapeutic window for chronic migraine prophylaxis but also allow intratumoural or convection-enhanced delivery in cancers where the blood–brain barrier, abnormal vasculature or acidic pH otherwise impede drug penetration. Early-phase clinical translation should therefore prioritise depot or nano-aerosol platforms capable of once-weekly or intranasal administration, respectively, to improve adherence over long preventive courses.

Small-scale, head-to-head trials already demonstrate that 3 mg immediate-release melatonin reduces headache days as effectively as 25 mg amitriptyline while offering anxiolytic and sleep-restorative advantages. These data justify exploring melatonin as an adjunct to gepants or CGRP-blocking antibodies to counteract the mild rise in insomnia and weight gain reported with chronic CGRP inhibition (62, 63). In oncology, pre-clinical gastric-, lung- and hepatocellular-cancer models reveal that pharmacological melatonin down-regulates tumour and myeloid PD-L1, remodels TAMs toward an IL-12 high/iNOS^+^ M1 phenotype, and boosts CD8^+^ T-cell infiltration—changes that synergistically enhance anti-PD-L1 checkpoint blockade. Strategically timing melatonin to precede checkpoint-inhibitor infusion by 2–3 h maximises MT-driven STAT-1 and NF-κB repression at the moment of antibody binding, a hypothesis now testable in window-of-opportunity trials that include migraine-burden endpoints.

Future studies should stratify participants by night-time salivary melatonin, MTNR1B polymorphisms, and tumoural BMAL1 expression to identify hypersensitive sub-populations. Wearable-derived circadian phase estimates and longitudinal CGRP or soluble-PD-L1 monitoring could serve as early pharmacodynamic read-outs, guiding iterative dose adjustments during prophylaxis or immunotherapy cycles. Decades of over-the-counter use support an excellent safety record, yet supraphysiological dosing (> 20 mg nightly) warrants ECG and endocrine surveillance in combination studies. Regulatory agencies increasingly view melatonin as a “platform modulator”; orphan-drug designations may therefore accelerate trials targeting glioma or intractable chronic migraine where standard options falter.

Key outstanding issues include (1) defining a ceiling dose that preserves immunostimulation yet avoids MT-receptor down-regulation, (2) dissecting interactions with glucocorticoid rhythms, and (3) resolving whether pulsatile (mimicking nocturnal spikes and preventing β-arrestin-mediated desensitisation) or continuous exposure (maintaining steady mitochondrial ROS quenching) optimally sustains benefit in trigeminovascular and tumour tissues. Multi-omic single-cell atlases across migraineurs and tumour-bearing hosts will be pivotal in mapping MT1/MT2 landscape shifts over disease course, while adaptive platform trials can expedite bench-to-bedside feedback.

Melatonin is poised to evolve from an ancillary sleep aid into a chronotherapeutic hub that tempers neurogenic inflammation and embeds circadian intelligence into cancer immunotherapy. Realising this potential will demand integration of precision-timed dosing, next-generation carriers and biomarker-guided co-medication strategies—an agenda that promises not only to mitigate migraine disability but also to unlock deeper, longer-lasting antitumour immunity.
